# Rapid Surface Charge Mapping Based on a Liquid Crystal Microchip

**DOI:** 10.3390/bios14040199

**Published:** 2024-04-18

**Authors:** Leixin Ouyang, Heyi Chen, Ruiting Xu, Rubia Shaik, Ge Zhang, Jiang Zhe

**Affiliations:** 1 Department of Mechanical Engineering, University of Akron, Akron, OH 44325, USA; lo10@uakron.edu (L.O.); hc77@uakron.edu (H.C.); rx7@uakron.edu (R.X.); 2 Department of Biomedical Engineering, University of Akron, Akron, OH 44325, USA; rs169@uakron.edu (R.S.); ge10@uakron.edu (G.Z.)

**Keywords:** surface charge mapping, liquid crystal, microfluidics, microchip, micropillar array

## Abstract

Rapid surface charge mapping of a solid surface remains a challenge. In this study, we present a novel microchip based on liquid crystals for assessing the surface charge distribution of a planar or soft surface. This chip enables rapid measurements of the local surface charge distribution of a charged surface. The chip consists of a micropillar array fabricated on a transparent indium tin oxide substrate, while the liquid crystal is used to fill in the gaps between the micropillar structures. When an object is placed on top of the chip, the local surface charge (or zeta potential) influences the orientation of the liquid crystal molecules, resulting in changes in the magnitude of transmitted light. By measuring the intensity of the transmitted light, the distribution of the surface charge can be accurately quantified. We calibrated the chip in a three-electrode configuration and demonstrated the validity of the chip for rapid surface charge mapping using a borosilicate glass slide. This chip offers noninvasive, rapid mapping of surface charges on charged surfaces, with no need for physical or chemical modifications, and has broad potential applications in biomedical research and advanced material design.

## 1. Introduction

Charged surfaces exist in many biological substances and various types of materials [1,2,3]. Characterizing the electrical charge distribution on these surfaces is critical for many applications. For example, the visualization of the cell membrane surface charge can be used for clinical cytological diagnosis [4,5]. Monitoring the charge distribution of tissues could predict its associated wound healing process [6,7]. The surface charge of materials enables their functions in directing cell attachment, mobility, proliferation, differentiation, signaling and protein adsorption [8,9,10,11]. Therefore, the rapid surface charge mapping of a solid surface can provide a powerful tool to facilitate the development of new biomarkers and the design of advanced materials [12,13,14,15,16]. 

To date, surface charge measurement on solid surfaces has remained a challenge. Electrophoretic methods, coupled with light scattering [17] or resistive pulse sensing [18], are used for the surface charge measurement of micro/nanoparticles. However, they cannot measure the surface charge of a solid surface. One can choose to measure the surface charge in terms of the streaming potential [19,20] of a capillary channel made out of the target surface. A pressure gradient applied to the capillary channel forces the electrolyte to flow through the capillary, inducing a streaming potential. As the streaming potential is a function of the surface charge (or zeta potential), the zeta potential of the microchannel surface can be estimated by measuring the streaming potential across the capillary channel. Although feasible in principle, the measurement setup is complex, requiring cutting the target surface into small pieces to form the capillary channel. More importantly, this method measures the average bulk zeta potential of the target surface with no mapping capability. Atomic force microscopy (AFM) [21,22,23], scanning ion conductance microscopy (SICM) [24,25,26], and photoelectrochemical imaging systems (PEISs) [27,28,29] can be used to measure the surface charge distribution of a surface. For AFM, when a charged AFM tip moves within the double diffusion layer (DDL) of the target surface, an interactive electrostatic force related to the charge density of the target surface is generated [30]. However, it is difficult to derive the local surface charge density from the force due to the difficulty in controlling the small tip–surface distance. In addition, mapping the surface charge requires scanning the entire surface by controlling the AFM tip movement, which is slow and challenging. Scanning ion conductance microscopy [24,25,26] is another method for surface charge mapping. When a single nanopipette is placed close to a charged surface, the ion current is changed due to the ion current rectification (ICR) effect [31,32], which is a function of the surface charge density [24,33]. However, for surface charge mapping, the nanopipette needs to approach the DDL of the surface first and return to a neutral position [26]. Thus, scanning the entire surface is labor-intensive and takes prohibitively long. PEISs use a focused laser beam to induce a photocurrent on an n-type semiconductor material, in which the local surface charge of cells can cause a photocurrent change [29,34]. Despite the surface charge mapping’s capability, it still relies on scanning the entire surface with the precise control of the focused laser beam movement point by point and, thus, is slow. 

Recently, Ouyang et al. [35] reported a method of cell surface charge mapping via a microelectrode array on an ITO surface. While this method achieved surface charge mapping of cells with a 4 μm resolution, it may not work for a hard surface, which may create scratches/damage on both the target surface and the microelectrodes. Further, the measurement resolution also needs improvement. 

To overcome the current challenges, we present a new method based on a liquid crystal-based microchip that can quickly measure the surface charge distribution of a planar surface (e.g., a glass surface or tissue surface). Liquid crystal is a state of matter that exhibits properties of both liquids and solid crystals. When the liquid crystal molecules are randomly oriented, the molecules have a certain degree of order, similar to that of a solid crystal. These molecules in a liquid crystal can still flow and change position, similar to a liquid. Also, liquid crystals are highly sensitive to external influences such as temperature, electric fields, and mechanical stress [36,37]. By manipulating these factors, it is possible to control the orientation and alignment of the liquid crystal molecules, leading to changes in their optical properties, such as light transmission, polarization, and refractive index [38]. Their unique properties, including tunable refractive indices and controllable light transmission, along with fast response times, low power consumption, and high optical quality, make them valuable for applications in imaging technologies. 

In addition, liquid crystals exhibit unique optical properties that can be used in sensing applications. In liquid crystal sensors, the orientation of the liquid crystal molecules is sensitive to external stimuli (e.g., electrical potential) which affects the opacity of the liquid crystal [38,39,40]. Liquid crystal sensors have several advantages, including high sensitivity, low cost, and simple manufacture. These advantages allow for liquid crystal-based sensors to be applied in many applications, including biomedical sensing, environmental monitoring, and industrial process control [38]. With this microchip, the object is placed on the top of a liquid crystal layer fabricated on a transparent indium tin oxide (ITO) substrate. An incident light transmits through the liquid crystal from the top of the microchip. The local surface charge/zeta potential of the object changes the orientation of liquid crystal molecules, causing a magnitude change in the transmitted light. Thus, the surface charge distribution can be quantified by measuring the intensity of the transmitted light. This method enables noninvasive and real-time surface charge mapping of a charged surface.

## 2. Materials and Methods

### 2.1. Detection Principle and Measurement Setup

**Figure 1:**
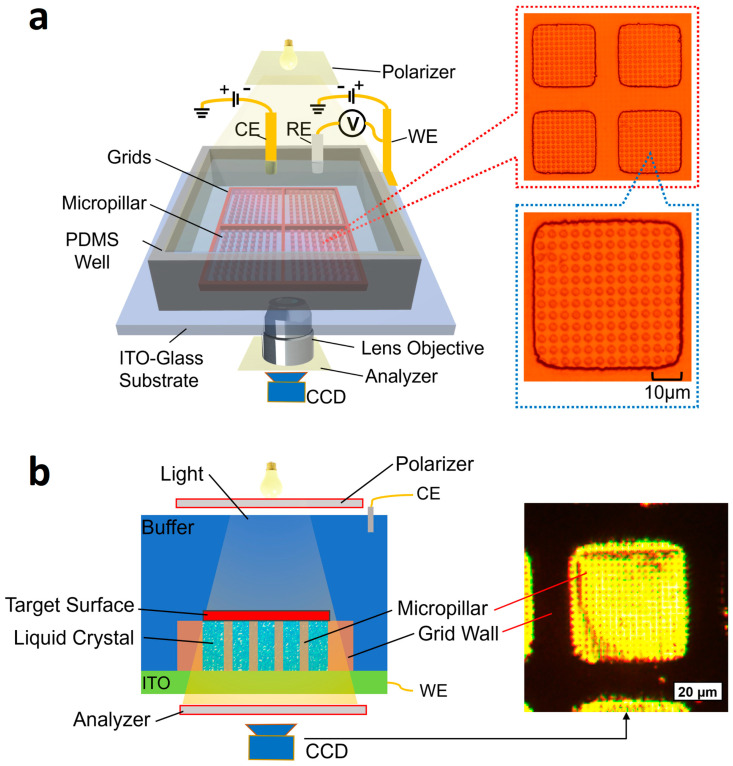
Illustration of rapid surface charge mapping of a planar surface using a liquid crystal-based microchip. The microchip consists of a micropillar array enclosed by microgrids. Liquid crystal fills in the gaps between the micropillar structures. An object sits on the surface of a micro support array. The incident light illuminates the liquid crystal from the top. The local surface charge of the surface induces a phase change of liquid crystal, which eventually creates a change in light transmission intensity. WE, RE, and CE are working, reference, and counter electrodes, respectively. A CCD camera captures the transmission light. (**a**) **Left**: the measurement setup of the microchip and enlarged images of the micropillars. **Right**: magnified images of micropillar array in microgrid wells. (**b**) **Left**: the side view of the microchip for surface charge mapping. **Right**: a microscopic picture of a micropillar array filled with liquid crystal.

Figure 1 illustrates the principle for rapid surface charge mapping of a planar surface using the liquid crystal microchip.

As shown in Figure 1, the microchip consists of the following components: (1) a micropillar array made of photoresist serving as the support for an object to be measured; (2) liquid crystal (Liquid crystal mixture E7, CAS 63748-28-7, BOC Sciences, Shirley, NY, USA) filling the gaps between the micropillar structures; (3) a photoresist micro grid to constrain the liquid crystal; and (4) a PDMS well to accommodate the counter and reference electrodes. The chip was fabricated on an ITO substrate, as shown in Figure 1a. Magnified images of the micropillar array in the microgrid wells are shown in the right column of Figure 1a. For surface charge mapping, an object is placed on the micropillar array, as shown in Figure 1b. In the right column of Figure 1b, a magnified image of a liquid crystal-filled micropillar array is shown. Each micropillar is designed to have a cross-section of 2 μm in diameter and a height of 2.5 μm. The gaps between the micropillars are 2 μm. The micropillar array has two purposes: (1) to prevent the target from sinking into the liquid crystal due to their weight, which could cause a change in the orientation of the liquid crystal molecules, and (2) to keep the liquid crystal surface relatively flat. Photoresist grids with a size of 42 μm (length) × 42 μm (width) × 2.5 μm (height) are designed as containers to prevent the liquid crystal from spreading out. 

A light beam was transmitted through the target and focused on the liquid crystal. In this configuration, the liquid crystal is sandwiched between the ITO and the buffer or the object. The ITO layer serves as the working electrode because of its good electrical conductivity and optical transparency. A polarizer and an analyzer are positioned near the top light source and the CCD camera, respectively, with their polarization directions perpendicular to each other. With the polarizer, only a light wave vibrating in a specific direction is allowed to pass through. Without the presence of liquid crystal, no light is observable by the CCD camera due to the perpendicular orientation of the analyzer. When the liquid crystal is introduced, the light is scattered in various directions; part of the light is passed through the analyzer and detected by the CCD camera. Furthermore, when a voltage change occurs between the WE and CE, the orientation of the liquid crystal molecules becomes more aligned in the vertical direction. Consequently, the intensity of the transmitted light varies accordingly. When a charged surface is placed on top of the chip, the local surface charge tends to cause a voltage change between the WE and CE. Thus, in principle, the local surface charge can be determined by measuring the transmitted light intensity. 

**Figure 2:**
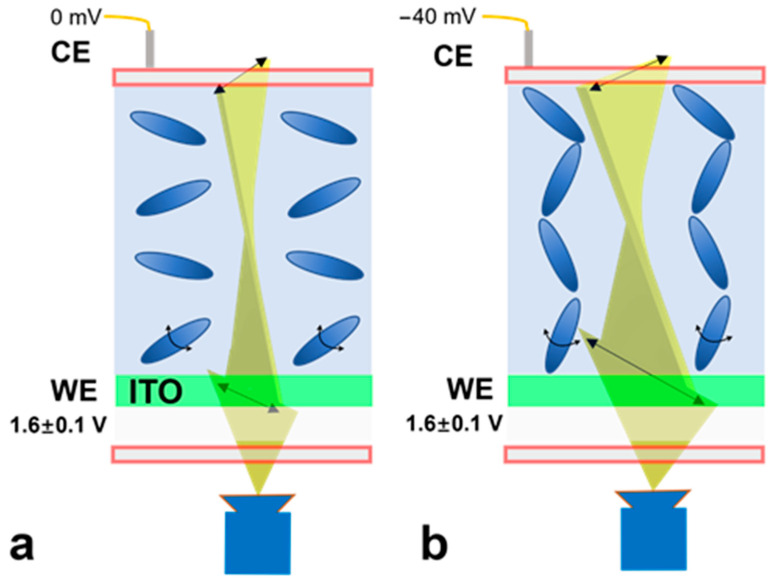
Schematic illustration of surface charge measurement mechanism. The working voltage for the ITO substrate (V_WE_) is 1.6 V (DC component) ± 0.1 V (square wave component, 1 Hz). (**a**) When zero voltage (V_CE_ = 0 mV) is applied to the microchip, the orientation of liquid crystal molecules is dispersed. The intensity of transmitted light is recorded as the baseline intensity. (**b**) When an electrical potential (e.g., V_CE_ = −40 mV, mimicking the zeta potential of a charged surface) is applied to the top of the microchip, the orientation of the liquid crystal is more aligned in the vertical direction, causing a change in intensity of transmitted light. Simultaneously, the AC excitation component (±0.1 V square wave) tends to twist the liquid crystal orientation. Upon a V_CE_ being applied (e.g., −40 mV), because the liquid crystal molecules are more aligned, they are less likely to be twisted by the AC component. Therefore, the peak-to-peak light transmission magnitude induced by the AC component decreases when the applied V_CE_ is decreased from 0 to −50 mV. The transmission light is recorded by a CCD camera; from the light intensity analysis, the local electrical potential can be backcalculated.

Figure 2 illustrates the surface charge measurement mechanism. First, an operation voltage (1.6 V (DC component) ± 0.1 V (square wave, 1 Hz)) is applied to the WE; the liquid crystal molecules are aligned in a dispersed direction. Once this operation voltage is varied by applying an electrical potential to the CE (V_CE_, e.g., −40 mV, mimicking a charged surface), the potential difference between the CE and WE increases. As a result, the liquid crystal molecules are aligned in a more vertical orientation, leading to the increased transmission of light (baseline shift). While this baseline transmitted light intensity can be used to calculate the applied electrical potential, the uneven thickness of the liquid crystal layer may cause the nonuniform distribution of light intensity. Simultaneously, the AC excitation component (±0.1 V square wave) tends to twist the liquid crystal orientation. Upon a negative V_CE_ being applied (e.g., −40 mV), because the liquid crystal molecules are more aligned, they are less likely to be twisted by the AC component. Therefore, the peak-to-peak light transmission magnitude induced by the AC component decreases when the applied V_CE_ is decreased from 0 to −50 mV; this magnitude of variation is not affected by the baseline. Hence, the peak-to-peak light transmission magnitude can be subsequently used to determine the applied external electrical potential. Note that the operation DC voltage applied to the ITO substrate (e.g., 1.6 V) is used to generate sufficient electrical potential to reorient the alignment of the liquid crystal molecules. The applied voltage needs to be kept as less than 2 V to prevent the generation of gas bubbles from electrolysis.

In short, the rapid measurement of the surface charge distribution of a charged surface is achieved by measuring the peak-to-peak magnitude of the transmitted light (M_PP_) through the liquid crystal, induced by a square-wave AC voltage. When no object is placed on the surface of the liquid crystal, the average intensity of the transmitted light remains unchanged. When a charged surface is placed on top of the microchip, it causes a local voltage potential change between the WE and CE, resulting in a variation in the peak-to-peak magnitude of the transmitted light intensity.

### 2.2. Device Fabrication

**Figure 3:**
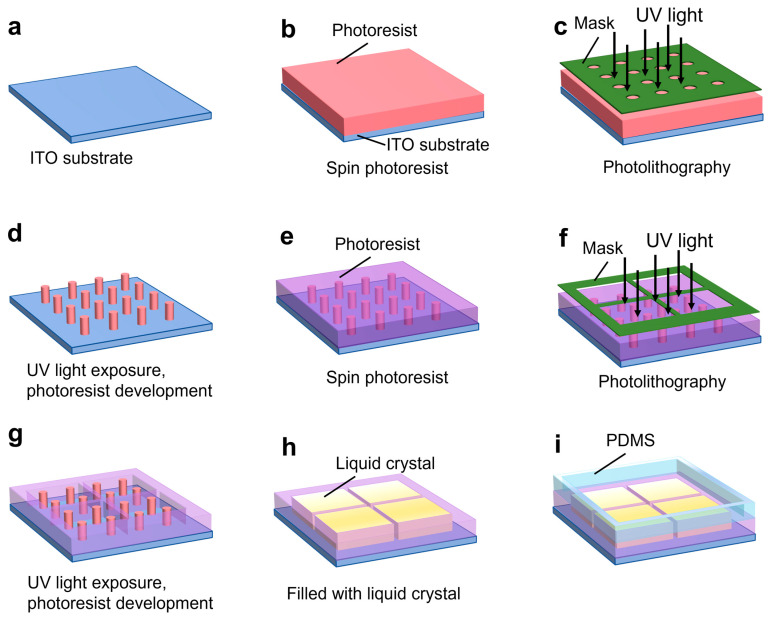
Illustration of the fabrication process flow for the microchip. Fabrication steps include (**a**) start with a bare ITO substrate, (**b**) spin-coat photoresist, (**c**) apply 1st photolithography, (**d**) photoresist exposure and development, (**e**) spin coat photoresist on micropillar array, (**f**) apply 2nd photolithography, (**g**) 2nd photoresist exposure and development, (**h**) fill in liquid crystal into microgrid, and (**i**) attach PDMS well.

The fabrication of the microchip is illustrated in Figure 3. The grids and micropillar were fabricated on a 2 inch-by-2 inch ITO substrate (Qunguan Electronic Technology Co., Ltd., Dongguan, China). The ITO layer thickness is 100 nm with a sheet resistance of 7 ohms/sq. For this application, a low-resistance ITO layer is necessary to allow for the voltage between the WE and CE to be applied across the liquid crystal layer. To fabricate the micropillars, the ITO substrate was coated with a 2.5 µm thick photoresist (AZ P4110), as shown in Figure 3b. UV light exposure was then applied, and the micropillars were fabricated by developing the photoresist (see Figure 3c,d). The substrate was placed on a hot plate for 2 h to consolidate the photoresist layer. To fabricate the grid wells, a second photoresist layer 2.5 μm thick (AZ P4110) was spin-coated onto the substrate with the micropillar (see Figure 3e). UV light was applied followed by photoresist development to fabricate the grid wells (see Figure 3f,g). The photoresist was consolidated again by a post-bake step. The grid container was then filled with liquid crystal; a thin flat glass plate was used to lightly press the liquid crystal surface in order to form a flat layer of liquid crystal (see Figure 3h). Finally, a 20 mm-by-20 mm PDMS well was fabricated using standard soft lithography and bonded to the ITO layer to facilitate the positioning of a target onto the liquid crystal (see Figure 3i).

A magnified micropillar array image is shown in Figure 1a. The top dimension was measured as 2.1 μm ± 0.2 μm in diameter. We also measured the thickness of the microgrid wall (2.53 ± 0.10 μm) using a Veeco 150 surface profiler (Veeco, Plainview, NY, USA) which has the same thickness as the micropillars. The microfabrication mainly consisted of standard photolithography; hence, the chip is cost-effective.

### 2.3. Materials and Experimental Setup

The three-electrode scheme was employed as shown in Figure 1. The ITO substrate (Qunguan Electronic Technology Co., Ltd., Dongguan, China) was used as the working electrode. The counter electrode was a commercially available platinum foil (Fisher Scientific, Waltham, MA, USA). The reference electrode was an Ag/AgCl electrode (Fisher Scientific, Waltham, MA, USA). 

The measurement setup of the microchip is illustrated in Figure 1. In the measurement, a (Fiber-Lite MI-150R, Dolan-Jenner Industries, Boxborough, MA, USA) light source was used to generate the incident light. The polarizer and the analyzer films (Renianus, Guangzhou, China) were used to only allow light with specific polarization to pass. Liquid crystal (Liquid crystal mixture E7 from BOC Sciences, Shirley, New York, NY, USA) filled the wells to sense the change in zeta potentials. The setup was placed under an inverted Olympus I-70 microscope (Olympus Life Science, Tokyo, Japan). In all measurements, we utilized a 20× magnification objective lens with a numerical aperture of 0.4. The transmitted light was detected by a CCD camera (MF603C-CCD from AmScope, Irvine, CA, USA). The captured images were subsequently processed using a MATLAB program to generate a peak-to-peak light-intensity map, which was then converted into a surface potential distribution.

### 2.4. Calibration

To calibrate the relation between the M_PP_ and the surface potential, the CE was placed very close (e.g., <1 mm) to the top surface of the liquid crystal. Hence, we can use the potential at the CE (V_CE_) to represent the external electric potential applied to the liquid crystal.

**Figure 4:**
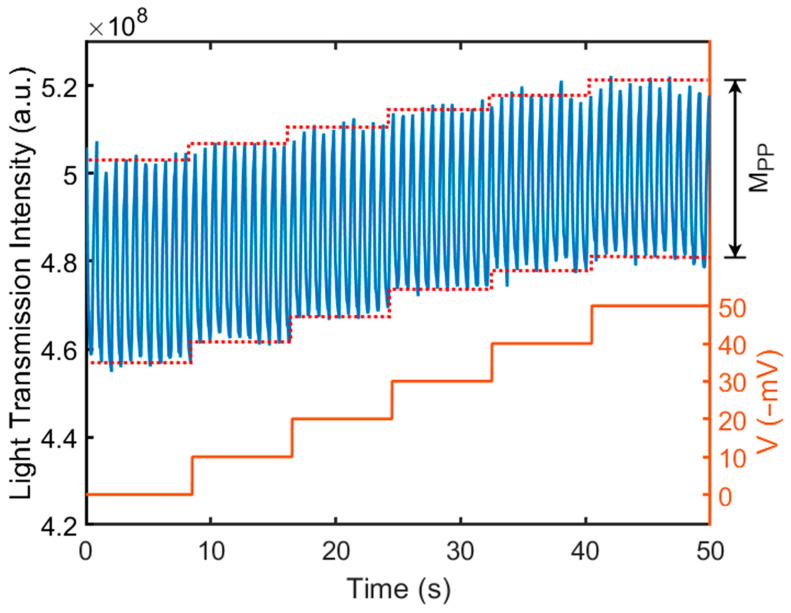
The intensity of transmitted light through a liquid crystal varies as a function of the applied electrical potential on CE. The electrical potential on CE was varied from 0 to −50 mV to represent the surface charge of an object. The change in the electrical potential on CE causes not only a change in transmission intensity of light, but also a change in the peak-to-peak transmission light magnitude, M_PP_. A CCD camera was used to capture the transmission light. The calibration was conducted in DPBS buffer solution.

**Figure 5:**
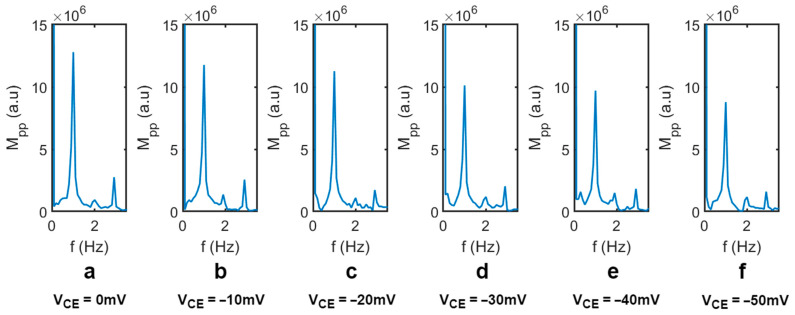
Typical transmission light peak-to-peak amplitude, M_PP_ (induced by the ±0.1 V AC component), analyzed by FFT (fast Fourier transform) when (**a**) V_CE_ = 0 mV, (**b**) V_CE_ = −10 mV, (**c**) V_CE_ = −20 mV, (**d**) V_CE_ = −30 mV, (**e**) V_CE_ = −40 mV, and (**f**) V_CE_ = −50 mV.

Figure 4 shows the transmitted light intensity through the liquid crystal varies as a function of the applied electrical potential on CE. Both RE and CE were placed in Dulbecco’s Phosphate-Buffered Saline (DPBS) solution (21-030-CV, Corning, Corning, NY, USA). A square-waved, external operation voltage of 1.6 V (DC) ± 0.1 V (square wave, 1 Hz) (V_WE_) was applied across the WE and CE. The frequency of the square-wave component was 1 Hz. Next, a DC electrical potential was applied to the CE (V_CE_), ranging from 0 to −50 mV. The V_CE_ represents the electric potential applied to the top surface of the liquid crystal. A change in the electric potential on the surface of the liquid crystal caused the orientation change of the liquid crystal molecules, resulting in the change in the peak-to-peak transmission magnitude (M_PP_), as shown in Figure 4. The transmitted light was collected by a CCD camera and was analyzed using fast Fourier transform (FFT). Figure 5 shows the peak-to-peak amplitude variation of the transmitted light intensity (M_PP_) at a different V_CE_ (from 0 mV to −50 mV) from the FFT analysis. As the V_CE_ decreased from 0 to −50 mV, it caused a variation in the applied voltage between the CE and WE, which resulted in a significant decrease in the M_PP_. Note that the V_CE_ was varied from 0 mV to −50 mV to mimic the zeta potential range of biomaterials and membranes in commonly used buffers [41,42,43]. We also conducted the measurement at a lower frequency, 0.5 Hz, with the V_CE_ varying from 0 to −50 mV; the light transmission intensity waveform and the resulting M_PP_ were nearly identical to those at 1 Hz. This confirmed the light intensity change is dependent only on the applied voltage amplitude.

**Figure 6:**
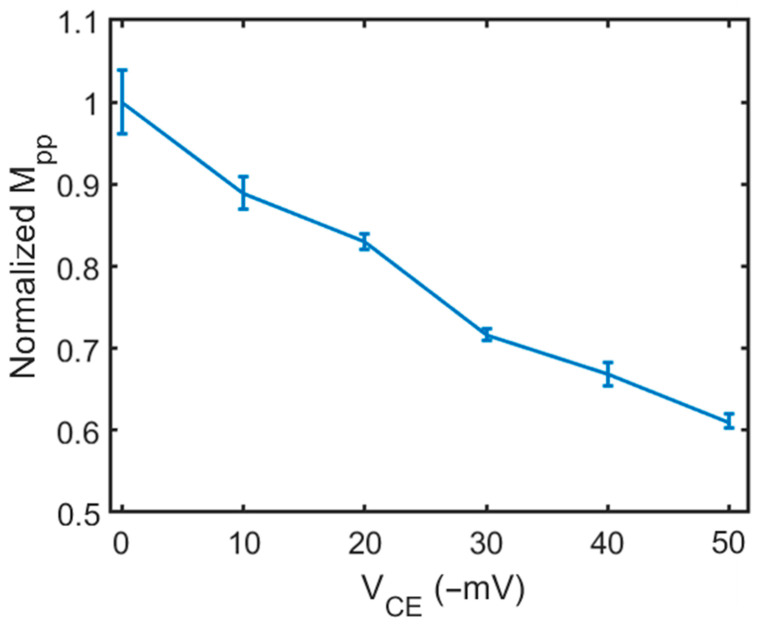
The calibration curve: the relation between the peak-to-peak transmitted light amplitude (M_PP_) and V_CE_ in DPBS buffer solution.

Next, we normalized the peak-to-peak transmission magnitude change (M_PP_) from Figure 5. First, at V_CE_ = 0 mV, the background M_PP_ value was measured and set as the baseline value. Subsequently, the M_PP_ was measured while the V_CE_ was varied from −10 to −50 mV; the resulting value was divided by the background intensity amplitude (baseline value), yielding the normalized amplitude. Figure 6 shows the relation between the normalized M_PP_ change and the electrical potential on the CE (V_CE_). The data points were connected using straight lines to represent the relationship. The error bar of each data point represents the standard deviation of five repeated measurements at each V_CE_. We also conducted the measurement on 5 different grid wells. The uncertainty is at a similar level. The relation is approximately linear. This calibration curve was used to measure the surface zeta potential of a planar surface in a DPBS buffer solution.

## 3. Surface Charge Mapping of a Planar Surface

**Figure 7:**
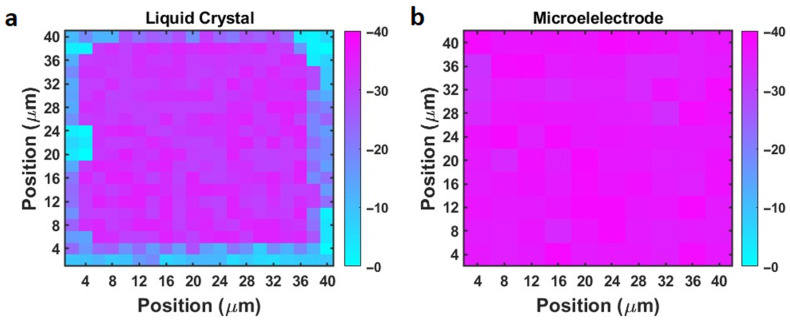
(**a**) The surface zeta potential mapping result of a borosilicate glass slide using the liquid crystal microchip. (**b**) The surface charge mapping measurement result of the same borosilicate glass slide using the microelectrode array method [35].

We measured the zeta potential of a borosilicate glass slide using the liquid crystal microchip. A small piece of borosilicate glass slide (5 mm × 5 mm × 0.1 mm (thickness)) was placed in DPBS buffer solution for 60 min, and then positioned on top of the microchip surface. The transmitted light intensities before and after the placement were measured. A post analysis was conducted to determine the peak-to-peak transmission amplitude (M_PP_) change of each 2 µm × 2 µm pixel, from which the local surface charge was determined. Figure 7a shows the typical mapping result, which was acquired in one microgrid well. In the data processing, the well area was partitioned into 2 μm-by-2 μm sub-squares (pixels) at the central point of each micropillar. The details of the pixel partition are illustrated in Figure S2 in the Supplemental Information. In each pixel, the micropillar area (no liquid crystal) only consists of 20% of the 2 μm-by-2 μm pixel. Note that we observed that the edge region of the liquid crystal microchip did not exhibit noticeable changes, while the central area exhibited a large change. In Figure 7a, the unresponsive region accounted for 27.5% of the micropillar region in this microgrid well. This ratio ranged from 25% to 35% for other functional microgrid wells. This could be attributed to the presence of photoresist residues near the edge of the grid, which created an insulating layer between the WE and CE. Hence, we analyzed the light intensity of the functional regions (central area) by excluding the nonresponsive regions, and backcalculated the local zeta potential. Figure 7 shows the mapping results. From the mapping, the average zeta potential of the glass slide was determined to be −31 ± 3 mV.

The photoresist residues at the edge were caused by the two-step photolithography shown in Figure 3. After the micropillar array was fabricated, we spin-coated a second layer of photoresist onto the micropillar structures. To avoid causing damage to the delicate micropillar structures during exposure, we intentionally left a gap between the photomask and the substrate, which could cause UV light scattering and leave underdeveloped photoresist at the edge. In addition, to mitigate the possible damage caused by the second exposure and second development of the micropillars, we reduced the exposure and development time of the photoresist; this also possibly caused photoresist residues at the edge. This problem can be mitigated by using one-step photolithography, i.e., the patterns of the micropillar array and the microgrids are all fabricated in one photolithography step.

To validate the measurement result, we also measured the zeta potential of the same glass slide using the microelectrode array method. The sensing principle and the measurement setup are described in a prior paper [35]. Figure 7b shows the mapping result. The average zeta potential was −35 ± 5 mV. The two sets of measurements by the microelectrode array and the liquid crystal microchip matched reasonably well within the uncertainty range. It is worth mentioning that because the ratio of the liquid crystal area to the micropillar surface area is large, a 2 μm × 2 μm mapping resolution can be achieved. 

The above measurement represented a rapid, noninvasive surface charge mapping method for any planar surface. The surface charge distribution can be quickly determined by measuring the transmitted light intensity without modifying the surface physically or chemically. The target surface is unlikely to be damaged due to the soft contact between the target surface and the liquid crystal. The mapping resolution is approximately 2 μm × 2 μm, which is higher than that of the microelectrode array method. While the demonstration was performed on a rigid planar surface mimicking the surface of materials, our plan for further experiments includes fabricating a micropillar array on a flexible ITO substrate to adapt this method for mapping nonplanar surfaces (e.g., tissue surface, etc.). In comparison, it is difficult to determine the local surface charge density from the force–distance curves when surface charge mapping using AFM because of (1) the existence of other forces (e.g., van der Waals interaction forces and short-range hydration forces [22]) and (2) the challenge of determining the distance between the AFM tip and the target surface. In comparison to scanning ion conductance microscopy for quantitatively mapping the cell surface charge, it also requires the positioning of a single nanopipette within 10 nm to 30 nm from the target surface [24,25,26], which is difficult to control. More importantly, scanning the entire cell surface via an AFM tip or a nanopipette is labor-intensive and time-consuming. Our liquid crystal microfluidic chip enables rapid surface charge mapping of the entire surface simultaneously, which can be performed with a typical microscope in a lab.

It is worth mentioning that this method is distinctively different from the microelectrode array method [35]. For the microelectrode array method, when a negatively charged surface is placed on the microelectrode array, the decreased local potential near the microelectrode enhances the local electrical field as well as the charge transfer from the solution to the microelectrode/ITO. Excess holes in the ITO are reduced, and more photons are absorbed. As a result, the intensity of the reflected light is reduced due to a surface electrical potential [35]. With the liquid crystal chip, the orientation of the liquid crystal molecules becomes more aligned, which results in an intensity change in the transmitted light through the liquid crystal. Compared to the microelectrode method, the liquid crystal method does not create scratches/damage on either the target surface or the microelectrodes.

## 4. Conclusions

We demonstrated a new surface charge mapping method for charged surfaces using a liquid crystal-based microchip. The microchip consists of a micropillar array enclosed in a photoresist grid fabricated on an ITO (indium tin oxide) surface. A charged surface suspended in electrolyte can be placed on top of the microchip. The local surface charge/zeta potential changes the orientation of the liquid crystal molecules, resulting in a change in the transmission light intensity. By measuring the transmission light intensity variations, local surface charges/zeta potentials can be obtained. A borosilicate glass surface was measured to demonstrate the chip. The zeta potential distribution result matched well with that measured by the microelectrode array method. This method/microchip works for the surface charge mapping of any planar surface. This rapid, noninvasive surface charge mapping method paved the way for analyzing the surface electrical properties of a variety of sample types including tissues and biomaterials as well as charged particles(e.g., virus, pollen, biomolecules)-adsorbed surfaces. Therefore, it can potentially benefit many critical areas, including disease diagnosis, functional biomaterials design, and environmental monitoring. 

## Data Availability

The data that support the findings of this study are available from the corresponding author upon reasonable request.

## Supplementary Materials

The following supporting information can be downloaded at https://www.mdpi.com/article/10.3390/bios14040199/s1, **Figure S1**: Images of micropillars (a) without liquid crystal and (b) with liquid crystal; Figure S2: Illustration of pixel partition from the center of each micropillar. Discussion on the effect of liquid crystal thickness; Discussion on ion conductivity of electrolyte.
