# Supplementary material for: Rapid Surface Charge Mapping Based on a Liquid Crystal Microchip
Source: Biosensors (Basel). 2024 Apr 18;14(4):199. doi: 10.3390/bios14040199 (PMC11047892; doi:10.3390/bios14040199)
Supplement: Supplementary file 1 [file biosensors-14-00199-s001.zip › biosensors-2930199-supplementary.pdf]

Supplemental Information

1. **Microscopic Images of micropillars** without and with liquid crystal

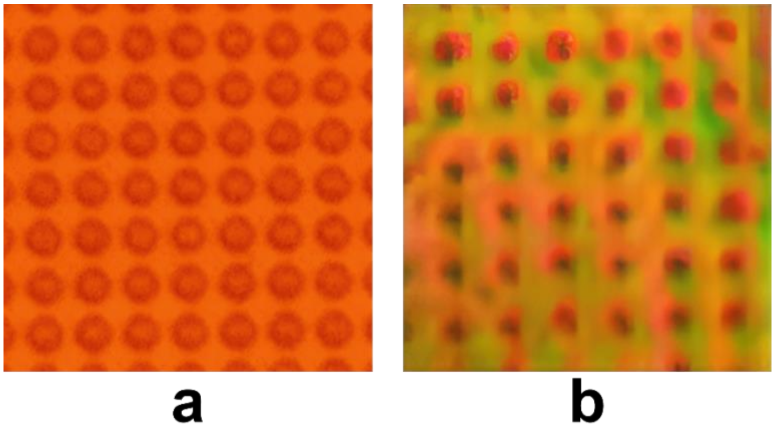

Fig. S1: Images of micropillars a) without liquid crystal and b) with liquid crystal.

Once the operation voltage (1.6 V (DC component) ± 0.1 V (Square wave, 1 Hz)) is varied by applying a $V_{CE,}$ (e.g. -40 mV), the liquid crystal molecules are aligned in a more vertical orientation, leading to increased transmission of light (baseline shift). Although this baseline transmitted light intensity can be used to calculate applied electrical potential, uneven thickness of the liquid crystal layer may cause nonuniform distribution of light intensity; this can be seen in Figure S1b, the liquid crystal color was somehow different around different pillars although the same V_{CE} was applied. This can potentially cause large measurement uncertainties if baseline shift is used to determine the surface charge. On the other hand, the ± 0.1 V square wave component tends to twist the liquid crystal orientation. Upon a negative V_{CE} is applied (e.g. -40mV), because the liquid crystal molecules are more aligned, they are less likely to be twisted by the AC component. Therefore, the peak-to-peak light transmission magnitude (M_{PP}) induced by the AC component decreases when the applied V_{CE} is decreased from 0mV to -40mV; this magnitude variation is not affected by the baseline shift. In this article, the peak-to-peak light transmission magnitude was used to determine the applied external electrical potential.

While we used the peak-to-peak transmission magnitude M_{PP} to determine the local electrical potential when V_{CE} varied from 0 to -40mV, we observed the change in M_{PP} was nearly unidentifiable by the naked eye; the change can only be obtained by post-processing. The post-processing is via FFT analysis; the results are shown in Figure 5.

2. **Illustration of pixel partition**

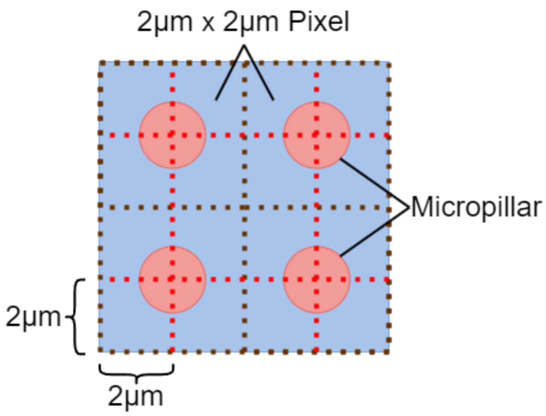

Fig. S2: Illustration of pixel partition from the center each micropillar.

In the imaging processing, the measurement area was partitioned into 2μm-by-2μm sub-squares (pixels) at the central point of each micropillar (illustrated in Figure S2). Because in each pixel, the micropillar area (with no liquid crystal) only consists of 20% of the 2μm-by-2μm pixel, the light intensity measured within the pixel can be used to represent the light intensity of the entire pixel. Therefore, the measurement can achieve a 2μm x 2μm resolution. Should smaller pixels be used (e.g., 1μm-by-1μm), some pixels (in micropillar area) would be primary blank pixels and have no response.

3. Discussion on the effect of liquid crystal thickness

The thickness of the liquid crystal is the same as the height of the micropillar structures (2.5μm). A thick liquid crystal layer may result in slower response times [https://opg.optica.org/ao/abstract.cfm?uri=ao-56-32-9050] due to the increased distance over which the liquid crystal molecules need to be reoriented under the applied electric field; higher voltage may also be needed. In our tests, we used a relatively thin liquid crystal layer (2.5μm) to improve the response time and reduce the operation voltage. Additionally, a thinner layer enhances optical performance by reducing light scattering and improving light modulation uniformity. On the other side, there are challenges to using a too-thin liquid crystal (e.g. 1μm). First, excess liquid crystals may be poured into shallow wells; removing extra liquid crystals is difficult and may stress the liquid crystal, which would change the orientation of the liquid crystal molecules and cause false positives. Secondly, we observed that a liquid crystal layer that was too thin tended to shrink to one droplet in a well during our preliminary tests. As the photoresist we used (AZ P4110) has a minimum thickness of 2μm, we chose 2.5μm to be the thickness.

4. Discussion on ion conductivity of electrolyte

DPBS was selected for the test because it is a buffer used for many bio-applications, and its conductivity is relatively high. In the 3-electrode calibration, the voltage applied on the counter electrode V_{CE} is used to represent the external electrical potential. If the ionic conductivity of the buffer is too low, the liquid resistance between the counter electrode and the liquid crystal surface may divide the applied voltage V_{CE} and cause errors in the calibration/measurement. While this can be overcome by shortening the distance between the counter electrode and liquid crystal surface, positioning the counter electrode extremely close to the chip (e.g., within ~100 μm) without damaging the structures on the chip is a challenge.
